# Medicinal plants cultivated in urban home gardens in Heredia, Costa Rica

**DOI:** 10.1186/s13002-022-00505-z

**Published:** 2022-02-12

**Authors:** Roxana González-Ball, Tania Bermúdez-Rojas, Marilyn Romero-Vargas, Melissa Ceuterick

**Affiliations:** 1 Universidad Nacional, Heredia, Costa Rica; 2 Health and Demographic Research, Ghent University, Ghent, Belgium

**Keywords:** Urban ethnobotany, Home gardens, Medicinal plants, Costa Rica

## Abstract

**Background:**

Urban ethnobotanical research in Costa Rica is rather rare and home gardens are poorly studied so far. Investigating their biodiversity is crucial in gathering knowledge on the uses of this particular flora, especially related to the owners’ health. This study therefore explores the diversity and knowledge of medicinal plants of private garden owners from three different urban neighborhoods in Heredia, Costa Rica, an thus far understudied area.

**Methods:**

Semi-structured interviews (n = 61) were conducted with garden owners in three socioeconomically different urban neighborhoods (Central Heredia, Maria Auxiliadora and Bernardo Benavides). Information was collected about medicinal plants cultivated in the garden, treatments, plant part used and mode of administration. All species were identified and their geographical origin was determined. This information was then compared with the available regional and local (ethno)pharmacopoeias to detect possible newly documented uses.

**Results:**

The majority or 90% of garden owners who also held knowledge on medicinal plants species were women﻿ (n = 30) of all ages (between 26 and 85 years old). A list of 27 species of medicinal plants was obtained from the participants of three urban neighborhoods. In Central Heredia, 74% (*n* = 20) of the total species were present, in Maria Auxiliadora 33% (*n* = 9) and in Bernardo Benavides 56% (*n* = 15). Most plant species were used by the participants to treat respiratory problems (11 spp.), hair and skin problems (9 spp.) and digestive disorders (8 spp.). Some plants were used to treat multiple ailments (10 spp.). About a third of all species (*n* = 8) were used by the participants to treat disorders that were not indicated in the regional and local pharmacopoeias. More specifically, *Aloe saponaria*, *Blechum pyramidatum*, *Costus scaber*, *Impatiens walleriana*, *Lippia alba*, *Tradescantia zebrina*, *Psidium friedrichsthalianum* and *Solenostemon scutellarioides* used for medicinal purposes by the participants were not found in the above-mentioned resources.

**Conclusions:**

The present study provides new information about the use of medicinal plants in a thus far understudied urban area in Costa Rica. We documented new medicinal uses for several plants listed in the regional and local pharmacopoeias as well as for plants not previously reported in an urban environment. In general, there is little information about the types of plants used for medicinal purposes in urban ecosystems in Costa Rica. Although the country has a high endemic diversity of plants, many exotic medicinal plant species were introduced by the Spaniards during the colonization and by Afro-Costa Rican descendants. The present results thus show how the diversity of the medicinal plants used by these garden owners' confirms a socioeconomic gradient and reflects both Costa Rica’s colonial history as well as the current epidemiological profile of the country. These findings underline the need for more ethnobotanical research in urban areas in Costa Rica.

## Background

There is a growing attention in ethnobiological literature to what Nabhan has called the ethnobiosphere in urban environments [1]. Urban home or domestic gardens are important indicators of medicinal plant use in the city [2, 3] and are therefore important sites to understand urban health practices and biocultural knowledge transfers [4, 5]. Medicinal plants form an important component of global health care as they are utilized as a main source of treatment in many developing countries and increasingly also in the global north [6]. Latin-America forms no exception to this trend, medicinal plants are widely employed in home medicine and phytotherapy in rural areas, often by indigenous and mestizo communities [4, 7], and in general, medicinal treatments form the second most important use category of local floras across the continent [8].

Urban ethnobotanical studies in Latin-America have so far mainly focused on (i) market studies [9–14] documenting the diversity of medicinal plant species sold, (ii) the dynamics of ethnobotanical practices of transnational and internal migrant communities [15–22] and to a lesser extent on (iii) urban home gardens. Home gardens have been studied in several Latin-American countries such as Mexico, Guatemala, Ecuador, Argentina and Brazil [23–27]. Some of these studies have explored cultivation of medicinal plants in different locations ranging from urban over suburban to rural home gardens [7, 28, 29], while others have focused more on identifying types of medicinal plant knowledge shared between rural and urban locations [23, 30]. In general, studies that focus on the floristic composition of home-gardens and other private green spaces often tend to report a relatively low percentage of species used for medicinal purposes compared to species used as food or ornamentals. A study in Nicaragua, for example [29], resulted in 293-total plant species, primarily used as ornamentals, although some medicinal plants were mentioned as well*.* A plant survey conducted in Bogota, Colombia, showed that a meagre 9% of the total plants registered were used medicinally, but only sparse information was given by the garden owners [31]. In addition, in a study in the Brazilian city of São Luís (Maranhão State) medicinal plants only represented 7.5% of the total [32]. Palheta and colleagues [27], on the other hand, found 127 medicinal species in the city of Abaetetuba in Brazil used as medicine. These varied findings not only point to a potential socioeconomic gradient in medicinal plant diversity and knowledge as we will describe further, but also indicate that documenting intra- and intercultural knowledge of medical plant diversity is much needed and also necessary to achieve conservation goals [33]. As such urban home gardens can contribute to both ecological, economic and social sustainability [8], which is ever so urgent in light of global climate change [6].

As a middle-income country with an advanced welfare and social security system, Costa Rica stands out from its neighboring countries in Central-America, for its excellent health outcomes [34], its economic and political stability and general social progressiveness [35]. In addition, Costa Rica has a long history of conservation of its exceptional biodiversity [36]. Despite the rich medicinal flora of the country, consisting of over 500 medicinal plant species (belonging to 104 genera)—of which 37% are harvested from their natural habitats and 126 are commercialized species [37, 38]—very little ethnobotanical research has been carried out, and even less so on the use of medicinal plants in urban areas. Ethnobotanical studies in Costa Rica have mainly focused on (i) specific species, growth forms or habitats [39–43] or on (ii) ethnobotanical practices within different indigenous communities [44–47]. However, to date, hardly any research has been conducted on the cultivation of medicinal plants in private urban domestic gardens and the knowledge surrounding their uses, other than the study by Madaleno and colleagues [48]. In the latter study, the authors compiled an inventory of medicinal plants used in San José based on interviews with urban gardeners, medicinal plant vendors from several local markets and a professional herbalist. Other research has only indirectly touched upon the use of medicinal plants in urban settings. García et al. [49] conducted interviews with patients at the Hospital Clinic in the capital San José, which revealed no less than 51 different plant species used for medicinal purposes.

The present study aims to close this gap in the existing urban ethnobotanical literature on Costa Rica by documenting the range of medicinal plants used by owners of urban private gardens (home gardens) in three socioeconomically different neighborhoods in Heredia (Central Heredia, Maria Auxiliadora and Bernardo Benavides). This is a previously unstudied area and thus provides new insights in the field.

## Methods

### Description of the study area

The study was conducted in the city of Heredia, located in the Central Valley, which is part of the greater urban area of Costa Rica, called the *Gran Área Metropolitana Region* (GAM). Heredia county has a total population of 19,138 inhabitants [50] in an area of just three km^2^ and has a population density of 6379.33 inhabitants per km^2^ which makes this the third largest city in Costa Rica, after the capital San José and Alajuela. With respect to the ethnic background of the inhabitants, most identify as white or *mestizo* (of mixed European and Amerindian heritage) in the population census of 2011 [51]. Heredia is a popular destination for economic migration within the country, as well as from the neighboring country Nicaragua [52]. With respect to the climate, the average annual rainfall is 2374.3 mm. The wettest months are September and October with average amounts of 410.88 mm and 424.6 mm, respectively [53]. A more detailed ecological description of the area can be found in [54].

### Selection of field sites

**Fig. 1:**
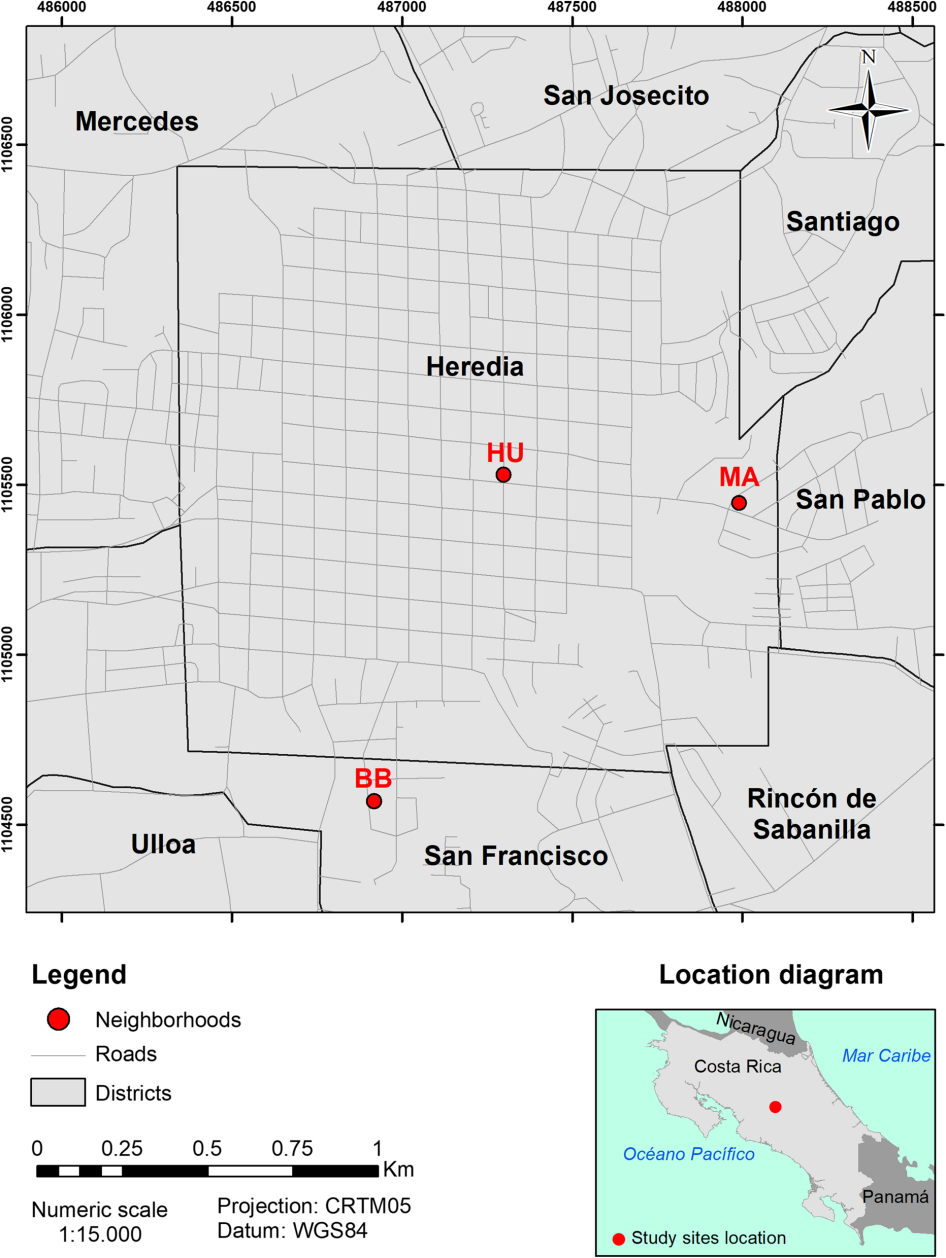
Location of the three neighborhoods where the urban private gardens were visited in Heredia, Costa Rica

Findings on the possibility of a socioeconomic gradient in biodiversity and ethnobotanical plant knowledge tend to differ across the literature [55–57]. Some studies have shown that more affluent urban neighborhoods tend to have a higher species richness, yet also show that these species are more often non-native, purely ornamental species, while garden owners from less affluent areas often cultivate more utilitarian, native species [55, 56] to provide food and medicines. A recent meta-analysis by Kuras and colleagues [57] offered a more nuanced interpretation to this dichotomous view. To avoid bias and to reach maximum variation and address possible intracultural variation in field sites and possible ethnobotanical practices, three types of socioeconomically different neighborhoods in Heredia were selected for the recruitment of urban domestic garden owners (see Fig. 1). We selected a mixed area characterized by commercial activities and housing (i.e. Central Heredia, here further referred to as UH), a residential area with a higher socioeconomic status (i.e. Maria Auxiliadora further referred to as MA) and a welfare housing development area (i.e. Bernardo Benavides further abbreviated as BB), as described in [58].

In the next step, gardens were identified through a stratified, random sample using Google Earth maps and the ArcGis software. The above information was later corroborated using a GPS to record the position of each garden. Subsequently, 10% of the total sample was selected for each category using SPSS. Each of the thus selected garden owners was contacted and invited to participate after providing information about the purpose of the research. If they did not agree to participate, another number from the generated random table was selected in order to complete the designated number of gardens devised for each of the three neighborhoods.

### Characteristics of the gardens

We defined domestic or home gardens as green areas (including backyards) that are located on the perimeter of a private property and contain plants that are grown in soil (and not in plant pots). This research was conducted only in urban domestic gardens whose owners lived in a home on the property, but did not take into account allotments or green areas of apartments or condominiums [54].

Prior visits to garden owners were made to obtain oral consent for participation. An information letter from the School of Biology at the Universidad Nacional de Costa Rica was presented to explain the purpose of the encompassing research project on urban private gardens by the first author [54, 58]. After giving oral consent and approval for the author to enter their property during their presence, garden owners were interviewed anonymously. The total sample consisted of 61 home gardens in the three types of neighborhoods [54, 58] with a total of 35 gardens in UH, 12 in MA and 14 in BB.

### Semi-structured interviews

To explore and describe garden owners’ knowledge and uses of medicinal plants in these Costa Rican urban gardens, a qualitative research design was followed after [59–61]. The semi-structured interview topic guide was validated during a pilot test which was conducted in a public park in the studied area with 50 garden owners who were asked to validate all questions [54]. The semi-structured questions focused on the floristic composition as well as on uses and management of the urban private domestic gardens [54]. Part of the results on the floristic diversity of these gardens has been previously published [58]. For the purpose of the current study, data were selected on cultivated medicinal plants and socio-demographic characteristics of the garden owners. Garden owners were asked which plants in their garden they used for medicinal purposes. We thus probed for active uses rather than for latent or passive knowledge [62]. Additional information was asked about the common name(s), exact treatment, used plant parts and preferred mode of administration. A total of 61 garden owners were interviewed between October 2011 and December 2012. They were selected based on garden ownership to validate that the correct information of each plant was given. General data about the 61 gardens, such as garden area, as well as gender and age of the participants, have been described extensively in [54, 58]. Of the 61 interviewed garden owners, 30 reported using and cultivating medicinal plants in their garden, corresponding to 12 for BB, 4 for MA and 14 for HU. The majority or 90% (*n* = 27) are female and only 10% (*n* = 3) are male. Their ages ranged from 26 to 85 years old (26–29 *n* = 2, 32 *n* = 1, 40–49 *n* = 2 and 50 ≥ years old *n* = 25).

### Identification of plant species

All medicinal plant species were identified in situ by the first author, who is a trained biologist with emphasis in botany. In addition, photographs were taken for the purpose of creating a digital voucher collection of the medicinal plants used in the studied gardens.

### Data analysis

Data obtained from the interviews were entered in a Microsoft Excel file in order to carry out descriptive statistics. Furthermore, information of the documented treatment mode of each medicinal plant was compared to the regional pharmacopoeias [63–71] in addition to the local Costa Rican literature on medicinal plants [72–75]. The comparison between fieldwork data and literature entries was based on the scientific names of the species used (see also [76]). In addition, relevant literature was reviewed in order to identify the geographical distribution of the medicinal plant species found.

## Results

### Plant species used

A total of 27 plant species (Table 1) used for medicinal purposes were found in the urban home gardens among the three different neighborhoods in Heredia (with UH *n* = 20 sp., MA *n* = 9 sp., BB *n* = 15 sp.). This is equivalent to 4% of the total floristic composition of 612 species of plants found in all studied gardens [54]. The main categories of uses include ornamentals (73%), plants without any specific use (15%) and fruits (6%) [54]. Table 1 provides an overview of the medicinal species used by the interviewed garden owners, including their Spanish common names, illnesses treated and neighborhood, information from the local pharmacopoeia, plant parts used, mode of preparation, use frequency and the origin of the plant species.

**Table 1:** Overview of medicinal species grown in home gardens in Heredia, Costa Rica

Family species	Neighborhood^a^	Spanish folk name	Origin	Treatment	Use documented in (local/regional) pharmacopoeia(s)	Plant part	Mode of preparation	Use frequency (%)
**Acanthaceae**								
*Blechum pyramidatum* (Lam.) Urb	BB	Sornia	Native	Treatment after radiation therapy for prostate cancer	Not reported, new use	Entire plant	Boil a bunch of the fresh plant and drink them over an 8-month period	3
*Peristrophe tinctoria* (Roxb.) Nees.i	HU	Azul de mata	Native	Hair dye	[75, 77]	Leaves	Boil several fresh leaves with stems in water and then apply to the hair after washing it	3
**Apiaceae**								
*Eryngium foetidum* L	BB	Culantro coyote	Native	Anaemia	[78, 79]	Entire plant	Boil a bunch of the fresh plant with beans, then drink the resultant soup	3
**Asparagaceae**								
*Aloe arborescens* Mill	MA,BB	Sábila	Africa	Skin allergies	[80, 81]	Leaves	Peel the leaves and rub the gel on the affected skin	3
*Aloe saponaria* Haw	BB, HU	Sábila	Africa	a. Digestive problems	a. [78, 79]	Leaves	a. Peel off the leaf, cut and add water to the gel and leave it in a pitcher for drinking	23
				b. For use on face burned skin	b. [82, 83]		b. Peel off the skin of the leaf and rub the geL	
				c. Lipoma (called fatty lumps)	c. Not reported, new use		c. Peel off the skin and put it on the affected area with a pad of chamomile tea	
*Aloe vera* (L.) Burm.f	BB,MA, HU	Sábila^b^	Africa	a. For refreshing and cleaning the colon, digestive problems	a. [67, 69]	Leaves	a. Peel off the leaf, blend it and leave it to rest for a while and drink it on an empty stomach	37
				b. Cleaning blood	b. [67, 84]		b. Blend the pulp with honey	
				c. Burned skin	c. [66, 67]		c. Peel off the leaf and rub the gel	
				d. Stomachache	d. [66, 69]		d. Blend the leaves with sugar	
				e. Earache	e. [85, 86]		e. Peel of the leaf, cut and add water and leave it in a pitcher for drinking	
							f. Put small pieces in the freezer and ingest them as a pill	
**Asteraceae**								
*Taraxacum officinale* F.H.Wigg	HU	Diente de león	Europe	Diabetes	[67, 77]	Leaves	Infusion	3
**Balsaminaceae**								
*Impatiens walleriana* Hook. f	BB	China	Asia	Healing wounds on the hands	Not reported, new use	Flowers	Rub the flowers on the hands	3
**Cactaceae**								
*Nopalea cochenillifera* (L.) Salm-Dyck	HU	Tuna	Mexico	Constipation	[77]	Leaves	Peel off the leaf, cut and add cold water and leave it in a pitcher for drinking	3
**Commelinaceae**								
*Tradescantia zebrina* Heynh	BB,MA	Cucaracha	Europe	High blood pressure = hypertension diabetes	Not reported, new use	Leaves, stems	Infusion: drink a small cup in the morning and at night	7
**Costaceae**								
*Costus scaber* Ruiz & Pav	MA,HU	Caña agria	Native	a. Urinary tract problems	a. [66, 87]	Stem	a. Infusion: macerated stem with corn hair and a fruit of *Psidium friedrichsthalianum* (*Cas* or Costa Rican Guava)	3
				b. Cough	b. Not reported, new use		b. Infusion with milk	
				c. Diabetes,	c. Not reported, new use		c. Macerate a stem in water for infusion	
**Euphorbiaceae**								
*Euphorbia tirucalli* L	BB	Lechosilla	Africa	Elimination of warts	[78, 88]	Leaf	Apply the milky sap from a leaf on the affected area only for external use	3
**Lamiaceae**								
*Mentha piperita* (Ehrh.) Briq	BB,MA,HU	Yerbabuena	Europe	a. Digestive problems	a. [66, 78]	Leaves, entire plant	a. Infusion using several fresh leaves with honey	17
				b. Intestinal worms (anthelminticum)	b. [66, 67]		b. Infusion in milk	
				c. Cough	c. [67, 88]		c. Infusion	
*Salvia rosmarinus* Schleid	MA,HU	Romero	Europe	a. Rheumatism, headache	a. [73, 74]	Leaves, entire plant	a. Mix several fresh leaves with rubbing alcohol	23
				b. Gum infections	b. [66, 89]		b. Boiled infusion with chamomile as mouthwash	
				c. External ulcers	c. [77]		c. Boil several fresh leaves and apply it to the skin	
				d. Muscular pain	d. [77, 90]		d. Mix the leaves with rubbing alcohol and camphor	
				e. Allergies	e. [73, 91]		e. Mode of preparation not mentioned	
				f. Fever	f. [67, 92]		f. Mix the leaves with rubbing alcohol and camphor	
				g. Hair loss (alopecia)	g. [73, 74, 77]		g. Boil the leaves in water and after washing the hair apply the liquid to the hair	
*Satureja viminea* L	BB,MA,HU	Menta	Europe	a. Stomachache, digestive problems	a. & b [79]	Leaves	a. Infusion with milk	13
				b. Stomach inflammation			b. Infusion boiled with leaves of *Lippia alba*	
*Solenostemon scutellarioides* (L.) Codd	BB	Chirrite	Asia	To stop bleeding of wounds (haemostatic)	Not reported, new use	Leaves	Put the adaxial (lower) part of the leaf over the wound	3
**Myrtaceae**								
*Eucalyptus cinerea* F. Muell. ex Benth	HU	Eucalipto	Australia	Respiratory problems	[63, 74]	Leaves	Infusion	3
*Pimenta dioica* (L.) Merr	HU	Jamaica	Caribbean Islands	Burning fat (losing weight)	[77, 93]	Leaves	Infusion	7
*Pimenta racemosa* (Mill.) J.W. Moore	HU	Bayrum	Caribbean Islands	Common cold	[94, 95]	Leaves	Infusion	3
*Psidium friedrichsthalianum* (O. Berg) Nied	HU	Cas	Native	Cough	Not reported, new use	Leaves	Boil several fresh leaves with mango leaves	3
**Poaceae**								
*Cymbopogon citratus* (DC.) Stapf	BB,HU	Zacate limón	Asia	a. Cough	a. [63, 73, 74]	Leaves	a. Strain the infusion before drinking (to remove hairs from leaves)	7
				b. Common cold	b. [63, 73, 74]		b. Boil several fresh leaves with leaves of *Psidium friedrichsthalianum* (*cas*) and add lemon before drinking it as a lemonade	
**Rutaceae**								
*Ruta chalepensis* (DC.) Stapf	MA,HU	Ruda	Europe	a. Sore throat	a. [96, 97]	Leaves, stems	a. Boil a bunch of the plant (leaves and stems) in water for gargling	17
				b. Earache	b. [63, 72, 74]		b. Heat some leaves and put them in the ear; add the leaves in a little bit of oil and then put this mix in a cotton ball and put in the affected ear	
				c. Common cold	c. [68]		c. Infusion with milk	
				d. Muscle pain	d. [66, 98]		d. Mix some leaves with rubbing alcohol	
**Scrophulariaceae**								
*Buddleja americana* L	HU	Salvia virgen	Native	For any illness (panacea)	Not reported as panacea	Leaves	Infusion	3
**Verbenaceae**								
*Lippia alba* (Mill.) N.E. Br. ex Britton & P. Wilson	BB,HU	Juanilama	Native	a. Digestive problems	a. [73, 74]	Leaves	a. Infusion of 5–6 fresh leaves	17
				b. Fractured bone	b. Not reported, new use		b. Apply *juanilama* tea on fractured leg	
				c. Stomachache	c. [73, 74]		c. Infusion	
*Lippia graveolens* Kunth	MA,HU	Orégano	Tropical America	Cough	[73, 74, 77]	Entire plant	Infusion with a piece of the plant (the size of the little finger) with several fresh leaves with milk	10
**Violaceae**								
*Viola odorata* L	BB,HU	Violeta	Europe	a. Gum infections	a. [69]	Roots, leaf	a. Boil the roots (decoction)	7
				b. Common cold	b. [69, 99]		b. Infusion of leaf	
**Zingiberaceae**								
*Zingiber officinale* Roscoe	HU	Jengibre	Asia	Cough	[79]	Root	Decoction then add honey	3

^a^UH: Central Heredia mixed area, MA: María Auxiliadora residential area; BB Bernardo Benavides welfare housing area

^b^The garden owners considered *Aloe vera* (L.) Burm. f. and *Aloe saponaria* Haw., both called *sábila*, to be the same species or a variety of the same species

**Fig. 2:**
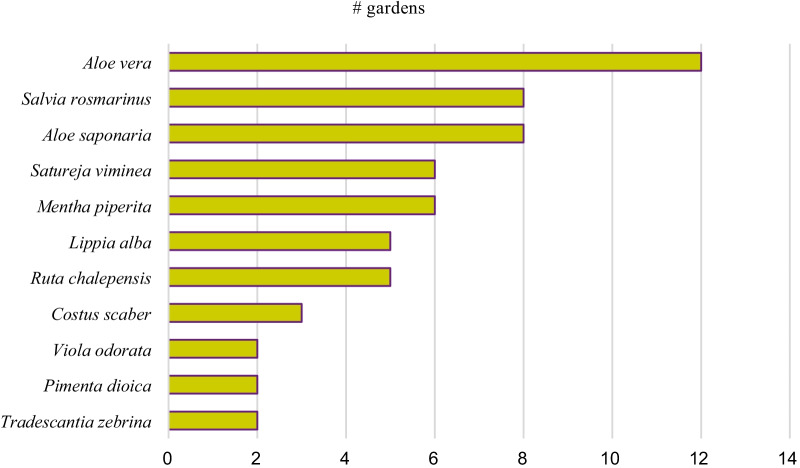
Species of medicinal plants found in at least two private urban gardens of Heredia

The 27 medicinal plants belong to 17 plant families. Lamiaceae and Myrtaceae are the most common plant families with each four species, Asparagaceae with three species followed by Verbenaceae with two species. All other species belong to different families. On species level, *Aloe vera*, *Aloe saponaria* and *Salvia rosmarinus* were reported most often by the participants (37%, 23% and 23%, respectively). Followed by *Mentha piperita*, *Ruta chalepensis* and *Lippia graveolens* (each used by 17% of the respondents). The rest of the medicinal plant species were used by less than 11% of the participants. Over a third of all medicinal species (*n* = 12) were found in at least two gardens. *Aloe vera*, *Aloe saponaria* and *Salvia rosmarinus* are among the more frequently cultivated medicinal species in the private gardens (Fig. 2). From the total number of medicinal species encountered, several of these species have been reported in San José before: *Mentha piperita* var. *citrata* and *M*. *spicata*, *Salvia rosmarinus, Aloe vera*, *Ruta graveolens*, *Cymbopogon citratus, Lippia alba, L. graveolens, Justicia pectoralis, Salvia rosmarinus, Mentha piperita, Eucalytus spp., Zingiber officinale, Ruta chalepensis, Tanacetum parthenium* and *Tradescantia zebrina* [48, 49].

In the area with the lowest socioeconomic status (BB), 86% of all garden owners reported at least one medicinal plant, in the mixed area (HU) 40% of all garden owners reported at least on medicinal species and in the residential area with a higher socioeconomic status (MA) only 33%. These findings confirm a socioeconomic gradient in the diversity of medicinal species and knowledge.

### Origins of plant species

**Fig. 3:**
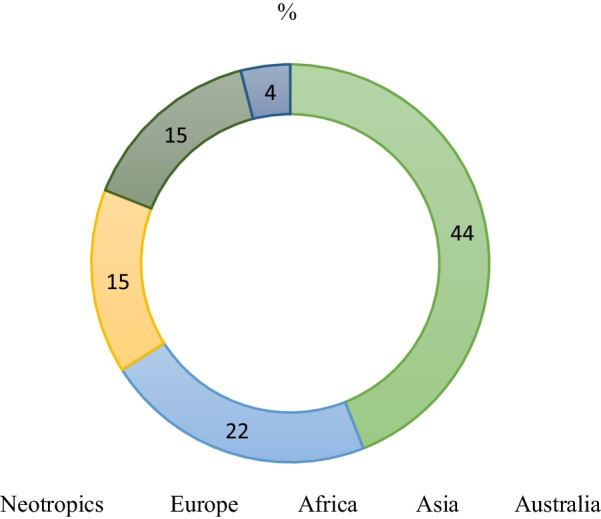
Natural geographical distribution of the medicinal plants found in the private urban domestic gardens in Heredia, Costa Rica

As shown in Fig. 3, only 33% of the species grown in these Heredian home gardens are native to Costa Rica (i.e., *Blechum pyramidatum*, *Justicia tinctoria*, *Eryngium foetidum*, *Tradescantia zebrina*, *Costus scaber*, *Nopalea cochenillifera*, *Psidium friedrichsthalianum, Buddleja americana* and *Lippia alba*), while 44% of all species are native to the Neotropics in general. The rest originated outside the continent, 37% of all medicinal species in this study have their origins in Europe and Africa, and 19% in Asia and Australia.

### Plant part used and dosage

As shown in Table 1, participants did not always indicate a specific dosage. Instead, they would often state that they use ‘several leaves’ or a ‘bunch of the plant.’ Leaves were the most commonly used plant part. This is unsurprising, as leaves are a tender part of the plant from which people can use simple extraction techniques [100], while fresh leaves produce larger quantities of active substances such as alkaloids, essences, glucosides and tannins [101] and are easy to use and available in major amounts throughout the year [27].

### Illnesses treated

**Fig. 4:**
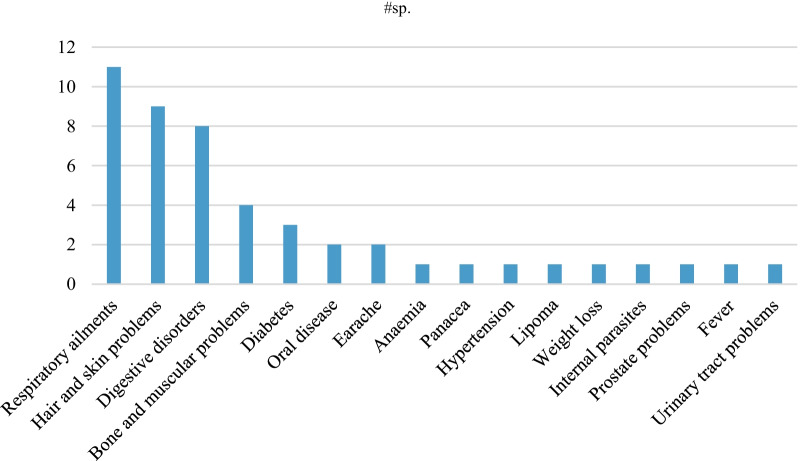
Number of medicinal plant species used per illness

Figure 4 shows the different illnesses treated and the number of medicinal plant species used by the participants. These clusters include emic illness categories and are categorized and presented here as they were mentioned by the participants. Most plants found in the urban gardens are used to treat respiratory problems (*n* = 11), hair and skin problems (*n* = 9) and digestive disorders (*n* = 8). These are all considered common, minor ailments, which are usually treated first with home-remedies in the hierarchy of the resort.

### Versatility

*Aloe saponaria, Aloe vera, Costus scaber, Mentha piperita, Salvia rosmarinus, Satureja viminea, Ruta chalepensis, Buddleja americana, Lippia alba* and *Viola odorata* were the most versatile species used for treating multiple illnesses.

### New uses

For the 27 species of plants found in this study, eight participants provided new information about treatments for various illnesses that differ from uses found in the regional and local pharmacopoeias. For the following plant species, there was a previously unreported use: *Blechum pyramidatum* (used after radiation therapy for prostate problems), *Aloe saponaria* (to treat lipoma, a non-cancerous tumor that is made up of fat cells also called fatty lump and also for earache), *Impatiens walleriana* (for healing wounds on the hands), *Tradescantia zebrina* (high blood pressure), *Costus* *scaber* (cough and diabetes), *Solenostemon scutellarioides* (haemostatic), *Psidium friedrichsthalianum* (cough) and *Lippia alba* (fractures).

## Discussion

### Diversity of medicinal plant species: garden owners’ characteristics

The majority of the garden owners that participated in this study is female. As Howard (2006) has shown in her exemplary research on gender and social dynamics in swidden and home gardens in Latin-America, it is not rare that more women are responsible for home gardens than men. Palheta and colleagues [27] also found that female family members are primarily responsible for taking care of urban home gardens in the Amazonian region of Brazil. Likewise, Duque [102] also found that much of the traditional medicinal knowledge in Colombian urban gardens is shared informally among women, but is also gathered by women who specialize in medicinal plants. In Latin-America, women are considered the keepers of traditional communal social relationships, food security and home health, and as such their participation in garden maintenance is key [8]. Moreover, zooming in on the participants' age, 83% of them was 50 years or older. This is in line with other urban ethnobotanical research that has shown that experience and knowledge of plants, especially medicinal ones, increases with age [103].

Compared to the total amount of plant species found in the overarching research project [58], the number of medicinal plant species is relatively low. Although other studies from urban areas in Mexico [32], for example, have shown comparable low proportions of medicinal species, compared to rural and suburban areas where the knowledge and use of medicinal plants is typically higher. The presence and composition of medicinal plant species in the studied gardens can be related to diverse factors, such as the personal preferences of the garden owners [104]. According to [32], garden owners managed these places based on tradition, needs and preferences, which means that they include species in their garden for diverse uses. The selection of the plant composition of a garden thus not only depends on the preferences of the garden owner but also on the alimentary, aesthetic and medicinal needs of the garden owners. Moreover, socioeconomic and cultural factors, as well as age and family situation also play a crucial role [104]. Which species garden owners cultivate and use is thus based on highly individualized and experiential knowledge as well as on their socioeconomic situation. As described above, scarce studies about the relation between specific medicinal plants cultivated by urban garden owners and their socioeconomic background seem to point to a socioeconomic gradient, also illustrated by our findings.

We assume that the relatively low number of medicinal plants found in the three areas might also be in part attributed to a low need, given the accessibility of medical care and hospitals located in their neighborhoods. The Costa Rican National Health Service created in 1941 provides reliable and almost universal access to health care [34] with little financial repercussions. Although the complementary use of medicinal plants in Costa Rica has been documented in other urban areas. People interviewed by [49] in a clinic in San José reported using medicinal plants in conjunction with prescribed or over-the-counter medicines without having any information about possible interactions.

### Origins of plants species: traces of Costa Rica’s colonial history

Finding exotic medicinal plants in these home gardens was not surprising, as many of these species were introduced during the Colonial Era. The Spanish Conquest of Costa Rica dates back to the sixteenth century. Soon people who were deported as slaves to the Caribbean coast from different African countries^1^ [105, 106] brought with them their own ethnobotanical practices and traditions. In addition to the mentioned Afro-Costa Rican descendants [37], there are several indigenous communities or *territorios indigenas* (Ngöbe, Bribri, Cabecar, Brunka, Maleku, Huetar and Chorotega) as well as people of European descent, which form a rich cultural mixture [107]. As a result, the current knowledge of medicinal plants in Costa Rica is a rich and hybrid mixture of knowledge from these different ethnic groups. These regional historical processes and cultural diversity are reflected in the medicinal plant uses in this study as well.

For example, *Aloe vera,* the species that is most frequently used by the participants in this study, was introduced to the Americas from the Canary Islands very early on during the Conquest. The species is now naturalized and found in numerous countries from Barbados, Jamaica, Antigua, Puerto Rico, Mexico to Central America, and as far as Texas, Florida and the Peruvian Andes [108]. *Aloe vera *is among the most used medicinal plant species reported by the participants and also belongs to most cultivated and consumed plants found in different Latin-American cities [7, 48]. In Costa Rica, there are between 60 and 80 hectares of *Aloe vera* used mostly as nutritional supplements [109].

Another frequently used species in this study, *Salvia rosmarinus* or rosemary, was among the first plants brought by the Spaniards during colonial times in Costa Rica in the sixteenth century [110] and still is frequently used today as confirmed in a survey carried out in an urban clinic in San José, Costa Rica [49].

In fact, it has been estimated that about a fourth of all plants used in traditional healing practices and domestic remedies in Costa Rica have been introduced by European colonizers [48], such as ginger *Zingiber officinale*, cloves *Pimenta racemosa*, Jamaican pepper *Pimenta dioica*, cinnamon *Cinnamomum zeylanicum* and aniseed *Pimpinella anisum* [111]. About half of the medicinal species grown in home gardens in San José, Costa Rica, are native to the American continent [112].

Similar results were found in other studies throughout the Neotropics. It is assumed that more than half of the herbs once utilized by Spanish colonizers were still cultivated and used all over Latin-America by the mid-twentieth century [113]. More recently, Bennett and Prance have estimated that nearly 80% of the introduced medicinal plant species that indigenous and mestizos of northern South America used are of European, Mediterranean or Asian origin [114]. These plants were initially introduced as ornamentals and food and were later used for medicinal purposes as well. Exotic medicinal plants have locally well-defined functions which suggests that they have a role in fulfilling specific local necessities that are not satisfied by native species [115]. Furthermore, the higher presence of exotic species of plants in urban gardens is related to the biotic homogenization process, where urbanization decreases the diversity and abundance of indigenous species in urban home gardens in Africa [116].

### Versatility and the hypothesis of diversification

The presence of many introduced medicinal species can be further explained by the versatility and diversification hypothesis that states that exotic plants are more likely to be introduced in traditional pharmacopoeias when fulfilling different purposes simultaneously [117]. Indeed most of the versatile species mentioned by the home garden owners to treat several illnesses are introduced (i.e., *Aloe saponaria, Aloe vera, Mentha piperita, Rosmarinus officinalis, Satureja viminea, Ruta chalepensis, Viola odorata)* with the exception of *Costus scaber, Lippia alba* and *Buddleja Americana*, which are native species. For the latter, no records were found in the pharmacopeia for its use as a panacea, only one participant expressed that it was used for any kind of illness. The observed versatility can be explained by the hypothesis of diversification, which suggests that exotic plants bring diversity to native species of medicinal plants already used, resulting in treatments that have a wider assortment of therapeutic goals and, in some cases, can treat illnesses that native flora cannot, thus providing an evolutionary benefit to the communities that incorporate them in the local pharmacopoeia [118, 119]. The versatility of introduced medicinal plants, in terms of the numerous conditions treated, may be the result of direct selection by healers or communities for more versatile plants that increase the opportunities for experimentation and, therefore, the probability of discovery of additional medicinal applications [117]. For example, the finding that the Lamiaceae are one of the families with most species in this research, might be related to the fact that many species in this family are used as both medicine and condiments and the importance of this family is mainly due to its species’ richness in essential oils that have widely known and studied medicinal properties [120]. This plant family also has the most species of exotic medicinal plants used by indigenous people of the northern part of South America [114].

### New uses for lifestyle diseases: a reflection of Costa Rica’s epidemiological profile

For the 27 medicinal species of plants found in this study, eight participants provided new information about treatments for various illnesses that differ from uses found in the regional and local pharmacopoeias [64–70, 72, 74, 107, 121]. Although some of these uses concern treatments for common minor ailments such as digestive problems and cough, other treatments involve uses for lifestyle diseases (hypertension, diabetes) and severe conditions (cancer, tumors). These latter types of uses might not be part of the shared common pharmacopoeia yet. Of the 4.301.712 million people in Costa Rica, 72.8% live in urban areas and 27.2% in rural regions [50] and although it is officially a developing nation it has an epidemiologic profile like most developed countries [34]. Costa Ricans have the second-highest life expectancy in the Americas, higher than the USA, Chile and Brazil [122]. According to the census of 2017, the main reason of mortality is related to cardiovascular diseases; secondly cancer and thirdly pneumonia and chronic lung diseases. Moreover, the three principal cardiovascular risk factors (survey 2014) are high blood pressure (suffered by 31.2% of the population), diabetes (14, 9%) and overweight and obesity (increasing from 62 to 77.3% in different age groups) [123–125]. This is in part attributable to a westernized lifestyle with a high intake of saturated fat and physical inactivity. These newly reported uses thus show how knowledge on medicinal plant species even in urban areas is resilient and creatively reapplied onto newly emerging lifestyle diseases. Hence, urban areas can be arenas where new medicinal knowledge is dynamically created, shared and reproduced. This also illustrates the need for more ethnobotanical studies in urban areas in order to preserve the traditional medicinal knowledge of people living in urban areas.

### A note on potential adverse uses

Finally, a concerning finding is that some participants did not specify the dosage they use (see also Table 1), although it is necessary to know the active ingredients of medicinal plants and the variety of their active ingredients in terms of their location in the plant (leaf, seed, stem, root) and their seasonal availability, in order to understand the benefits and risks of using them [100]. In addition, some adverse uses were found. According to Rodriguez^2^ (pers. comm., 2012) *Satureja viminea* should not be used for medicinal purposes because of this species can affect the liver and kidneys. For verification, more toxicity tests should be conducted since pulegone, a major essential oil component, has been noted to have hepatotoxic function [126]. Another finding that contrasts with existing literature concerns *Nopalea cochenillifera* which is used for constipation by participants in this study, although its medicinal use has been contested [127].

## Conclusions

There is little information about medicinal plants from urban areas in Costa Rica and especially about plants cultivated by urban private garden owners. This research provides information about the medicinal plants used in selected urban areas in Heredia, Costa Rica. Some of the medicinal plants found had other uses than those found in the local and regional pharmacopoeias and others had no previously reported use at all. Several plant species are used to treat several different illnesses. Most plants are used to treat minor ailments, such as respiratory problems, digestive disorders, damaged hair and skin problems by the participants located in this urban area. The majority of the plants were introduced and versatile species, which is most likely related to the cultural mix existent in Costa Rica interconnected with the colonial heritage. It has been suggested before that the Costa Rican government should develop projects to rescue popular plants used for medicinal purposes according to ethnomedical traditions [74]. We agree as stated by TRAMIL [128] that the conservation estate of medicinal plants should be approached not only from a biological perspective, but also from a sociocultural perspective and, as such, should result in a better understanding of the dynamics that involve the ethnobotanical uses of these plants. Validating the correlations of the ethnomedicinal uses, bioactive substances, biological and pharmacological effects is of special importance and is a primary task for future research. Efforts are also needed to investigate the physiological and biochemical functions demonstrated by these understudied species, identifying the individual bioactive natural products and illustrating their mechanisms of action. Hence, we recommend that further studies should be conducted in other urban locations within Costa Rica to add to the current local knowledge of what plants are being used for medicinal purposes.

## Figures and Tables

**Fig. 5:**
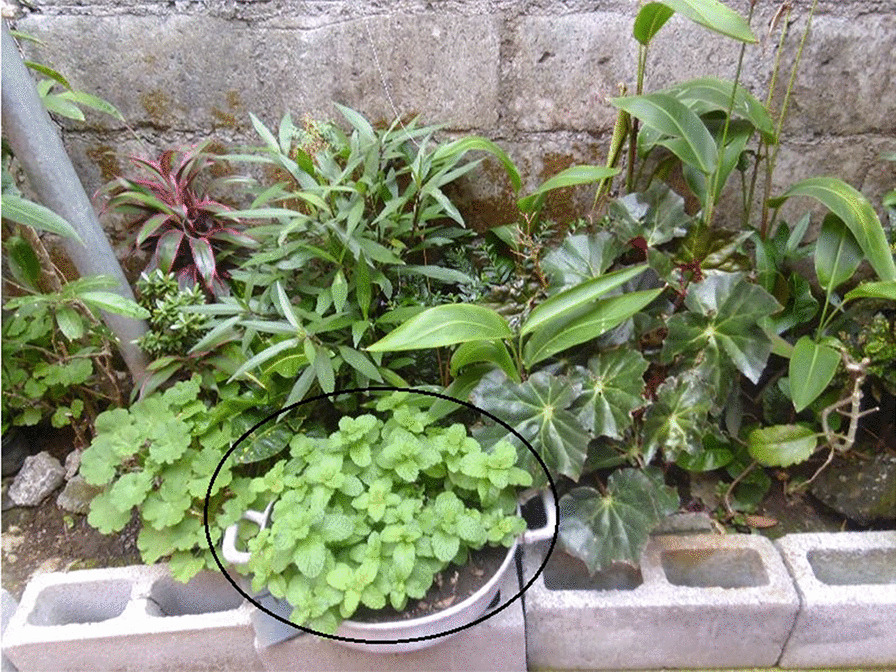
*Mentha piperita* (HU)

**Fig. 6:**
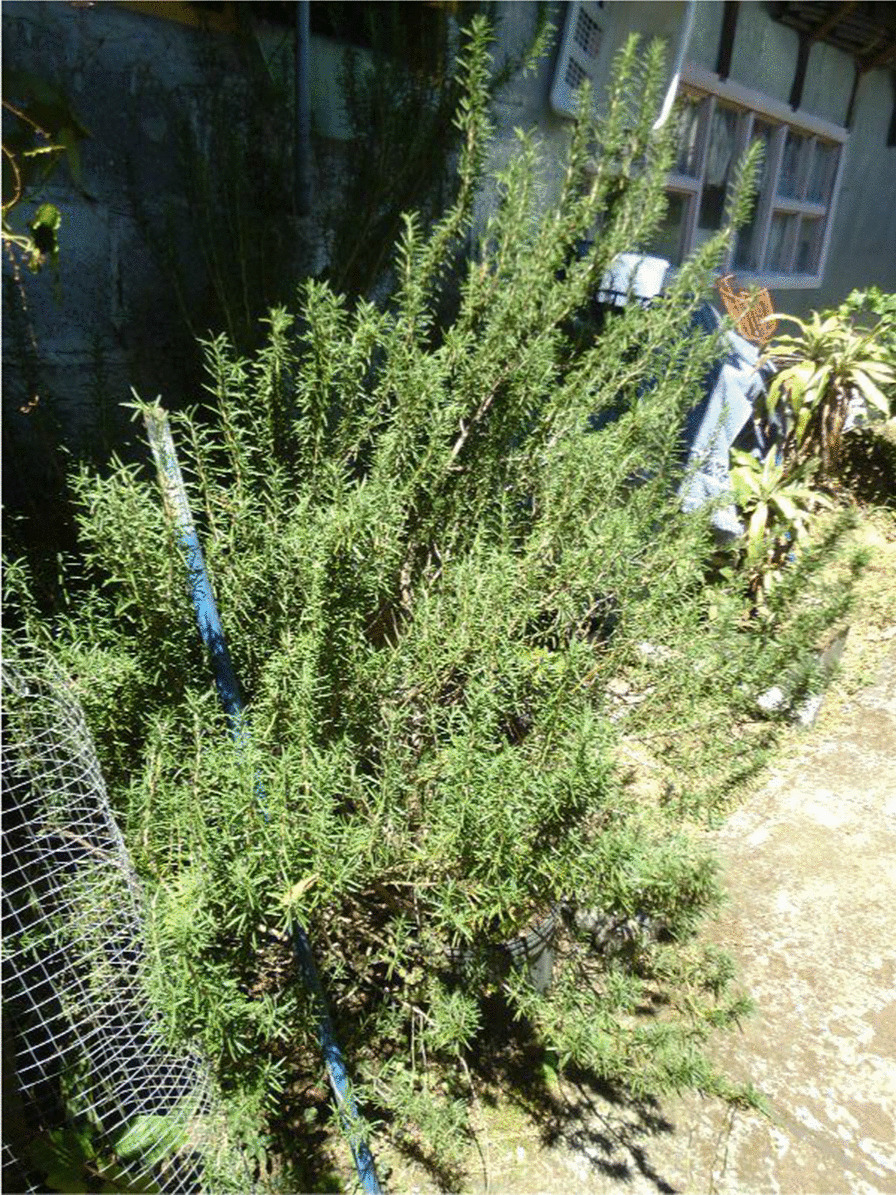
*Rosmarinus officinalis* (HU)

**Fig. 7:**
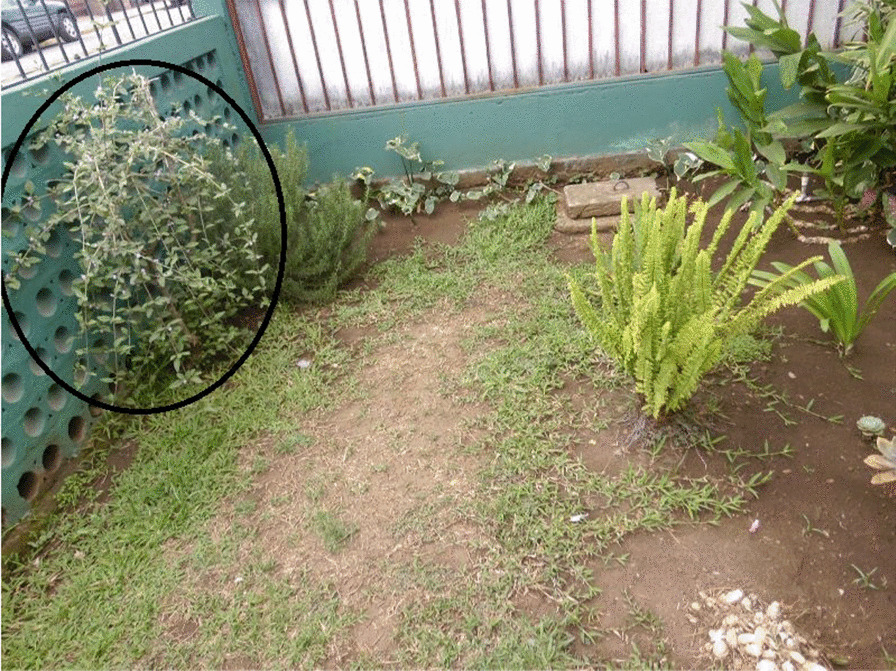
*Lippia alba* (HU)

**Fig. 8:**
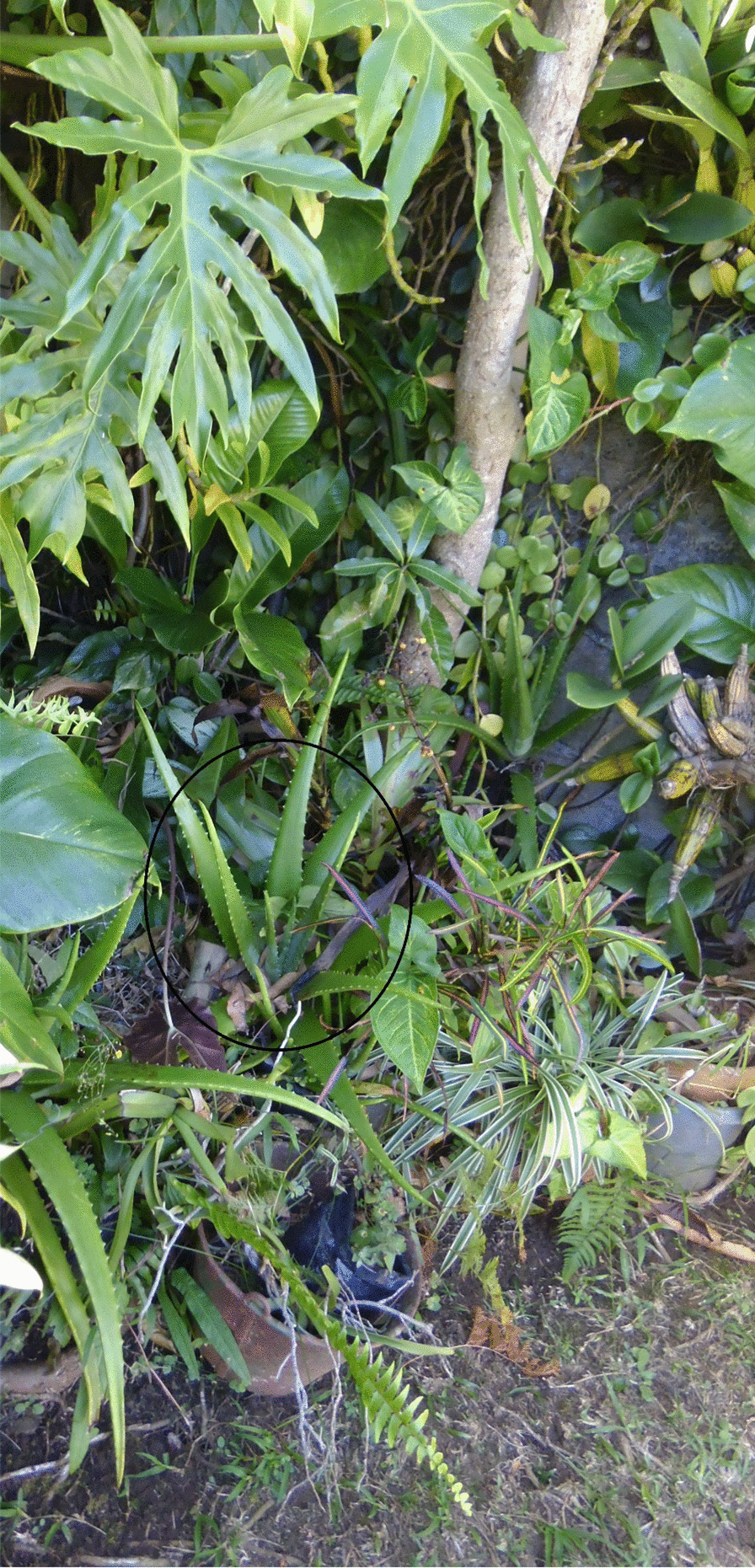
*Aloe vera *(MA)

**Fig. 9:**
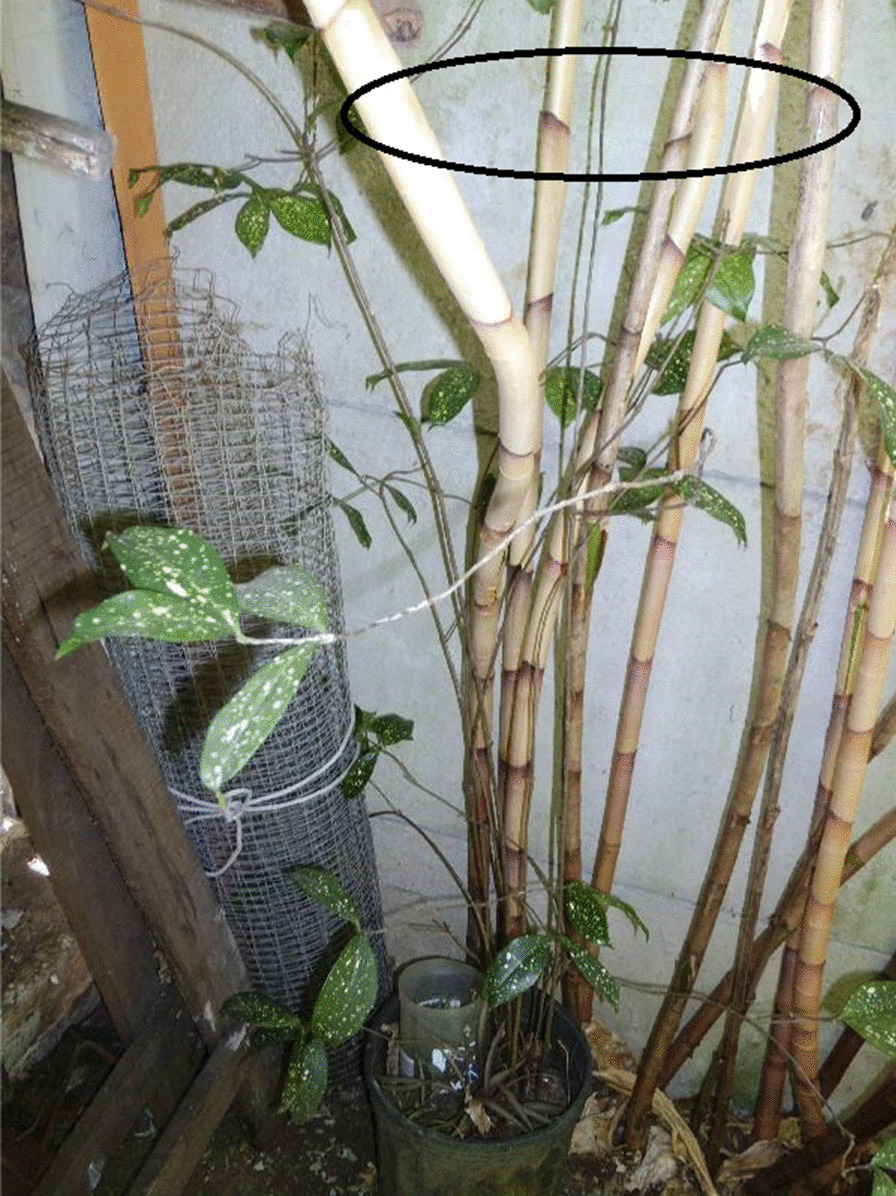
*Costus scaber* (MA)

**Fig. 10:**
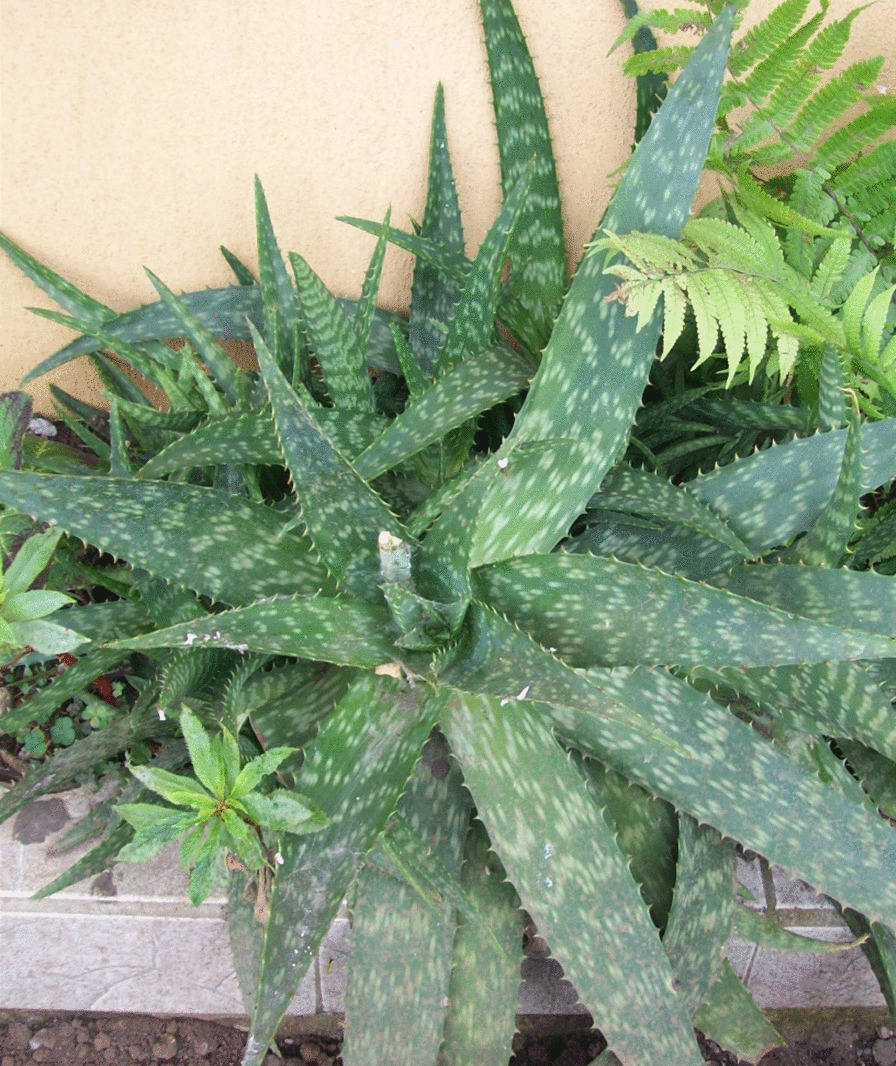
*Aloe saponaria *(BB)

## Data Availability

Please contact author for data requests.

## Appendix

See Figs. 5, 6, 7, 8, 9, and 10.

## Footnotes

1 The first historic registration of African slaves in Costa Rica dates back to the year 1522–1523 when 30 African individuals were brought by Gil González during his first excursion to the Pacific coast of Costa Rica and Nicaragua, followed by nine others that came with Hernán Sánchez de Badajoz in 1540. By 1569 Monsignor Thiel reported in the population census of Costa Rica a total of 17,479 individuals, of which 30 were described as ‘black’ people and 170 were described as ‘Mulatto,’ ‘Zambos’ and ‘Pardos.’

2 Hernán Rodríguez is a Costa Rican agronomist and ethnobotanist who currently teaches workshops on the use of medicinal plants for the general public and universities. The first author had assisted in some of his courses.
